# Population Cancer Risks Associated with Coal Mining: A Systematic Review

**DOI:** 10.1371/journal.pone.0071312

**Published:** 2013-08-15

**Authors:** Wiley D. Jenkins, W. Jay Christian, Georgia Mueller, K. Thomas Robbins

**Affiliations:** 1 Center for Clinical Research, Department of Family and Community Medicine, Southern Illinois University School of Medicine, Springfield, Illinois, United States of America; 2 College of Public Health, University of Kentucky, Lexington, Kentucky, United States of America; 3 Center for Clinical Research, Southern Illinois University School of Medicine, Springfield, Illinois, United States of America; 4 Simmons Cancer Institute, Department of Surgery, Southern Illinois University School of Medicine, Springfield, Illinois, United States of America; Universität Bochum, Germany

## Abstract

**Background:**

Coal is produced across 25 states and provides 42% of US energy. With production expected to increase 7.6% by 2035, proximate populations remain at risk of exposure to carcinogenic coal products such as silica dust and organic compounds. It is unclear if population exposure is associated with increased risk, or even which cancers have been studied in this regard.

**Methods:**

We performed a systematic review of English-language manuscripts published since 1980 to determine if coal mining exposure was associated with increased cancer risk (incidence and mortality).

**Results:**

Of 34 studies identified, 27 studied coal mining as an occupational exposure (coal miner cohort or as a retrospective risk factor) but only seven explored health effects in surrounding populations. Overall, risk assessments were reported for 20 cancer site categories, but their results and frequency varied considerably. Incidence and mortality risk assessments were: negative (no increase) for 12 sites; positive for 1 site; and discordant for 7 sites (e.g. lung, gastric). However, 10 sites had only a single study reporting incidence risk (4 sites had none), and 11 sites had only a single study reporting mortality risk (2 sites had none). The ecological study data were particularly meager, reporting assessments for only 9 sites. While mortality assessments were reported for each, 6 had only a single report and only 2 sites had reported incidence assessments.

**Conclusions:**

The reported assessments are too meager, and at times contradictory, to make definitive conclusions about population cancer risk due to coal mining. However, the preponderance of this and other data support many of Hill’s criteria for causation. The paucity of data regarding population exposure and risk, the widespread geographical extent of coal mining activity, and the continuing importance of coal for US energy, warrant further studies of population exposure and risk.

## Introduction

Cancers are several of the leading causes of death in the US, and disparities persist in both incidence and mortality. The American Cancer Society (ACS) and the National Cancer Institute (NCI) report that one in every four American deaths is attributable to cancer. The ACS estimates 901,230 new cancer diagnoses and 279,710 cancer deaths in the US are attributable to cancers at the four most common sites: female breast, colorectal, lung and bronchus, and prostate (B/C/L/P) [1]. According to the NCI Cancer Trends Progress Report, improvements in personal lifestyle behaviors, such as smoking, nutrition and physical activity could reduce cancer deaths by 50–75 percent [2]. However, a disproportionate cancer burden exists among people who cannot reduce their risk by personal choice. While the overall mortality and incidence rates for cancer are declining in our country, certain populations continue to show higher risk and worse outcomes in cancer-related illness (e.g., blacks are more likely to develop and die from cancer, and be diagnosed at a later stage, than other races and ethnicities) [3].

A complex set of economic, geographic, and social determinants of health create cancer health disparities. Some risk factors, such as age and family history, are largely due to biological mechanisms and the accumulation of risks and exposures over time and cannot be modified [4]. However, there remain potentially modifiable risk factors to which individuals may be exposed without their knowledge, and to disparate levels based upon race and location [5]. Location is particularly relevant when considering exposure to industrial operations. While there are studies showing increased cancer risk due to occupational exposure to carcinogens, there is a paucity of data examining the impact of industrial operations to the cancer rates of potentially exposed surrounding populations (non-occupationally exposed) [6]–[8]. The potential for such exposure is large, as the United State Environmental Protection Agency Enforcement Division pursued 1,754 civil and 64 criminal cases for violation of the federal Clean Air and Water Acts in 2012 alone [9]. The health effects to the surrounding populations are largely unknown, and it is important to determine if proximity to specific industries is associated with increased cancer risk so that appropriate protective measures may be taken. This is of increased importance for industries which are of large scale or great geospatial extent, and thus present increased potential for widespread exposure.

**Figure 1:**
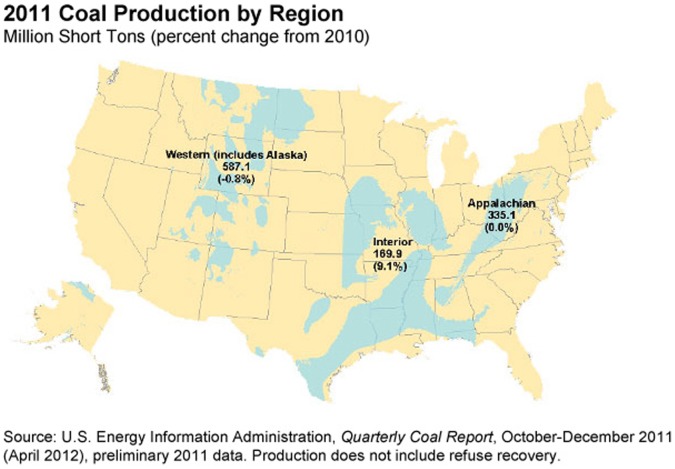
Distribution of coal-bearing stratifications in the 48 contiguous United States.

In recent years the extraction of fossil fuels has attracted substantial attention for its potentially damaging effects to the environment and human health [10]–[12]. The US mined >1 billion tons of coal in 2011, with 90% being used for domestic electricity production in 580 coal-fired power plants [13]. Coal fuels nearly half (42%) of the 4 trillion kilowatt-hours of electricity generated in the United States in 2011 [14]. Coal is produced in 25 states across three major coal-producing regions (see Figure 1). US production is estimated to increase 7.6% by 2035, and current production rates result in an estimated coal reserve exceeding 200 years [14], [15]. Oil and gas reserves at the global level are estimated to be sufficient through 2100, but there is risk to US national security in reliance upon foreign sources of power. For example, 22% of imported petroleum comes from the Persian Gulf states and another 11% from Venezuela [16]. These circumstances, and the development of more effective scrubbing mechanisms and other technological advances, have resulted in sustained interest in coal as a source of fuel (especially for large-scale electrical generation). As coal mining both continues and expands in large areas of the continental US, it is therefore important to understand the health risks potentially associated with such activity so that preventive measures may be adopted as needed. The geospatial extent of coal bearing fields is considerable (Figure 1), underlying, for example, 33% of Missouri and 68% of Illinois [17].

Our objective was to review recent peer-reviewed literature to assess the evidence of a relationship between exposure to coal mining activities and cancer incidence and mortality. We will thus explore studies relating to several specific cancer sites, as well as different sources and routes of exposures, to identify gaps or weaknesses in the literature where future research may be profitably directed.

## Methods

Eligibility criteria included: English language, peer-review, publication since 1980, basis in human subjects, and explicit examination of coal mining and associated or subsequent cancer of any kind. Furthermore, we excluded studies focusing on investigations of radiological associations with cancer as they are not specific to coal mining. We searched PubMed, EbscoHost (Academic Search Premier and MEDLINE Complete), and Cochrane Library using the terms: ‘cancer’ in TITLE and ‘coal mining’ in ANY FIELD as well as ‘coal mining’ in TITLE and ‘cancer’ in ANY FIELD. Retrieved articles’ bibliographies were reviewed for additional manuscripts not otherwise identified. The authors reviewed study abstracts retrieved by the search and determined eligibility by consensus. We extracted the following information from each study, as available: study design, population studied and size, exposure type, reported cancer end points and strengths of association (e.g. OR, RR, SMR), and statistical significance (e.g. *p*-values or confidence intervals).

There are several risks of bias at the level of the individual study. For example, occupational studies of coal miners may suffer from ‘healthy worker effect” whereby only those individuals who are of greater health engage in this physically demanding profession – resulting in a lower estimate of risk compared to the general population [18], [19]. On the other hand, coal miners are subject to a considerable number of long-term studies of health and health outcomes – resulting in perhaps greater surveillance and identification of disease than experienced by the general population. Bias at a larger scale may be due to publication bias present in the underreporting/under publishing of studies showing no association between coal mining and cancer.

## Results

**Figure 2:**
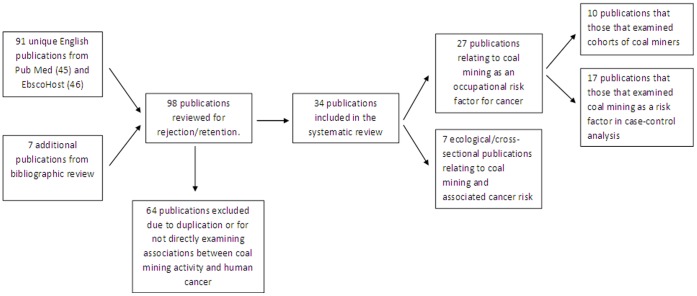
Article identification, review, and retention flowchart.

The initial search criteria returned 98 unique manuscripts (45 from PubMed, 46 from EbscoHost, and additional 7 from bibliographic review). All abstracts were reviewed by WDJ and GM for inclusion based upon study criterion and rejection/retention determined by consensus. From these, 64 were removed as not directly examining associations between coal mining activity and human cancer or duplicate reporting of study results (Figure 2). The remaining 34 studies were separated into two main categories: A) 27 studies of coal mining as an occupational risk factor for cancer [19]–[45], and B) 7 ecological/cross-sectional studies of coal mining and associated cancer risk in the surrounding population [46]–[52]. The occupation studies may be further classified as: A1) those that examined cohorts of coal miners (standardized incidence/mortality ratios calculated; SIR/SMR; relative risks (RR)) [19]–[28], and A2) those examining coal mining as a risk factor in case-control analysis (odds ratios (OR) calculated) [29]–[45]. While categories A1 and A2 both explicitly examine coal mining and associated cancer risk, category A1 does so by specifically selecting coal miners for comparison to others (e.g. cohort studies) while category A2 includes coal mining as a risk factor. Table 1 lists all retained studies and important information from each. Tables 2 and 3 describe the cancer risks drawn from these studies. As studies were performed over differing time periods and in differing places, there was some inconsistency in how cancer was reported and we have thus condensed results of studies of similar cancers into single categories (e.g. ‘gastric’ and ‘stomach’ into the category ‘Digestive/Gastric/Stomach’). The evidence presented by these studies is both inconsistent in that some examine incidence, others mortality, and some both, as well as frequently contradictory in the direction of the results.

**Table 1:** Manuscripts identified by the systematic review and stratified by A1) occupational cohorts, A2) occupational risk factors, and B) ecological population-level studies.

	Authors	Year	Location(s)	Subjects	Exposure(s)	Outcome(s)	Study Design	Findings
**A1**	Acheson et al.	1981	England & Wales	Miners and quarrymen	Occupation	Nasal cancer incidence	Cross-sectional	Significantly higher incidence (SIR) of nasal cancer was observed for coal miners.
	Attfield & Kuempel	2008	U.S.	8899 working coal miners from 31 mines	Coal mine dust, coal region	Stomach, lung cancer mortality	Cohort	No convincing evidence of associations between dust exposure and stomach, lung cancers
	Atuhaire et al.	1986	Wales	7939 men, miners and non-miners	Occupation	Gastric cancer mortality	Cohort	No evidence that coal mining is associated with gastric cancer mortality.
	Brown et al.	1997	New South Wales, Australia	Male coal industry workers (n = 23,630)	Occupation, stratified by mine type– underground vs. “open-cut”	Incidence of all cancers, several specific cancers	Cohort	“No evidence of serious hazard for cancer in modern coal mines…” (p. 32)
	Kuempel et al.	1995	U.S.	9078 male coal miners from 31 mines across the U.S.	Coal mine dust	Stomach, lung cancer mortality	Cohort	No evidence of associations between dust exposure and stomach, lung cancers
	Miller & Jacobsen	1985	England	Approximately 25,000 British coalminers	Occupation	Digestive system cancer mortality	Cohort	Compared to other men in coal mining regions of England and Wales, coal miners had elevated risk of deaths from cancers of the digestive system (mostly stomach cancer). No increase observed for lung cancer among coal miners.
	Miller & MacCalman	2009	Great Britain	17,820 British coal workers	Coalmine dust and quartz dust	Lung and stomach cancer mortality, among others	Cohort	Lung cancer mortality was associated with exposure to coal mine dust with high quartz content
	Morfeld et al.	1997	Germany	4578 coal miners in Saar region of western Germany	Occupation	Stomach cancer, lung cancer mortality	Cohort	No elevated risk observed for lung cancer, some evidence for increased risk of stomach cancer.
	Swaen et al.	1995	The Netherlands	3790 coal miners with abnormal chest x-rays	Occupation, years of employment	Disease-specific mortality, including cancers	Cohort	Among coal miners with chest x-rays indicating coal workers’ pneumoconiosis (CWP) or other pulmonary pathology, there was excess mortality from gastric cancer.
	Tomaskova et al.	2012	Czech Republic	Former coal miners with and without CWP	Diagnosis with CWP	Lung, stomach, bladder, colon, kidney cancer incidence	Cohort	Significantly elevated risk of lung cancer was found for former coal miners with CWP.
**A2**	Ames	1983	U.S.	184 coal miners from NIOSH cohorts database	Coal dust, years of underground mining	Gastric cancer incidence	Case-control	Elevated risk of gastric cancer was observed for smokers only.
	Ames & Gamble	1983	U.S.	184 coal miners from NIOSH cohorts database	Years of underground mining, pulmonary function	Stomach cancer, lung cancer incidence	Cohort	For miners with airway obstruction or long-term smoking, coal mine dust exposure was associated with stomach cancer risk
	Ames et al.	1983	U.S.	317 white male lung cancer deaths from NIOSH cohorts	Years of underground mining	Lung cancer mortality	Case-control	No evidence of an association between years of underground mining and lung cancer.
	Coggon et al.	1990	England	95 cases and 190 controls	Coal mining occupation and length of employment	Stomach Cancer incidence	Case-control	Cases were not significantly more likely to have been coal miners.
	Cordier et al.	1993	France	1530 cases and controls recruited from seven hospitals	Occupation	Bladder cancer incidence	Case-control	Odds of employment in coal mining and exposure to coal dust were significantly higher among those with bladder cancer.
	Goldberg et al.	1997	France	528 cases, plus 305 controls recruited from 15 hospitals	Occupation	Incident cancers of hypopharynx, larynx	Case-control	Significantly elevated risk of cancers for miners and quarrymen, including coal mining specifically.
	Golka et al.	1998	Germany	826 men from an area of former coal, iron, steel industries	Occupation	Bladder cancer incidence	Case-control	After adjustment for cigarette smoking, a significant risk of bladder cancer was observed for hard coal miners.
	Gonzalez et al.	1991	Spain	354 cases, plus 354 controls	Occupation	Gastric cancer incidence	Case-control	Significantly increased risk of gastric cancer observed for coal mining workers.
	Hosgood	2012	China	260 lung cancer cases, 260 age-matched controls (all farmers)	Occupation, years of employment	Lung cancer incidence	Case-control	Significantly higher risk of lung cancer in coal miners compared to controls.
	Jockel	1998	West Germany	1004 cases and 1004 matched controls recruited from hospitals	Occupation	Lung cancer incidence	Case-control	No increased risk of lung cancer.
	Lloyd et al.	1986	Scotland	42 lung cancer cases and 42 matched controls	Occupational exposure to dust	Lung cancer incidence	Case-control	No evidence of significant associations noted.
	Meijers et al.	1988	The Netherlands	381 cases and 381 controls recruited from one hospital	Occupation, stratified also by type of mining	Lung cancer incidence	Case-control	No evidence of significant associations noted.
	Schifflers et al.	1987	Belgium	74 bladder cancer cases and 74 matched controls	Occupation	Bladder cancer incidence	Case-control	Coal miners had higher risk of bladder cancer, but results were not quite statistically significant.
	Swaen et al.	1987	The Netherlands	683 male cases and 683 controls	Duration of coal mining occupation	Gastric cancer	Case-control	No evidence of significant associations noted.
	Swanson et al.	1993	U.S.–Detroit metropolitan area	3792 lung cancer cases and 1966 colorectal cancer controls	Duration of coal mining as occupation	Lung cancer incidence	Case-control	Significant trend in lung cancer risk observed for increasing years of employment in coal mining, but individual ORs marginal.
	Une et al.	1995	Japan	1796 coal miners, 4022 non-miners	Occupation	Mortality from several cancers, all cancers combined	Cohort	Coal miners had significantly higher risk of any cancer, and those with 15+ years of experience had significantly higher risk of lung cancer.
	Weinberg et al.	1985	U.S.–four counties in PA	176 cases, plus three control groups	Occupation	Stomach cancer incidence	Case-control	Cases were not significantly more likely to be coal miners or wives of coal miners.
**B**	Christian et al.	2011	U.S.–KY	Population-based	Coal mining activity	Lung cancer incidence	Ecologic	Several counties with high coal mining activity had significantly elevated lung cancer risk after adjustment for lifetime smoking prevalence.
	Davies	1980	Great Britain	Population-based–residents of 10 towns in Nottinghamshire	Residence in mining towns	Stomach cancer mortality	Ecologic	No evidence of significant associations noted.
	Fernandez-Navarro et al.	2012	Spain	Population-based	Proximity to underground and open pit mines	Cancer mortality	Ecologic	High mortality for some types of cancer, including lung cancer, colorectal cancer, bladder cancer, and leukemia, was associated with proximity to coal mining activity.
	Hendryx et al.	2008	U.S.–Appalachia	Population-based	Coal mining activity by county	Lung cancer mortality	Ecologic	Lung cancer mortality was elevated in Appalachian counties with heavy coal mining activity.
	Hendryx et al.	2010	U.S.–WV	Population-based	Proximity to coal mining industry	Mortality due to several cancers	Ecologic	A newly-developed “distance-weighted, at-risk population coal mining exposure measure” computed using a GIS was highly correlated with breast, respiratory, other, and total cancer rates of census block groups.
	Hendryx et al.	2012	U.S.–WV	773 adults in two rural communities, one in a mountaintop mining area	Proximity to coal mining industry	Self-reported cancer incidence	Cross-sectional	Self-reported incidence of cancer was elevated in coal mining areas after adjusting for other factors.
	Minowa et al.	1988	Japan	Population-based	Coal mining activity in local administrative units	Lung cancer mortality	Ecologic	Higher lung cancer mortality observed in administrative units with coal mines.

**Table 2:** Estimations of cancer risks reported from occupational studies (both occupation as cohort and case-control risk factor).

Cancer	Increased risk?	Values^reference^	N	Caveats
Bladder	Yes	OR = 2.42 CI 1.25–4.67^33^	N_c_/N_con_ = 765	Odds ratio for risk of bladder cancer; males 1984–1987; hospital based case-controlled
		OR_M-H_ = 2.54 CI 1.64–3.93^35^	N_c_/N_con_ = 412, 414	1984–1989; Odds ratio for risk of bladder cancer; adjusted for smoking; M-H = Mantel-Haenszel test
	No	SMR = 35 CI 16–66, P<0.05^27^	N = 3,790	Significant only those w/no/mild pneumoconiosis; inverse pneumoconiosis grade and cancer
		SIR_w_ = 0.95 CI 0.48–1.69^28^	N_w_ = 2,158	Ex-miners with pneumoconiosis only; smoking data incomplete
		SIR_w/o_ = 0.72 CI 0.44–1.10^28^	N_w/o_ = 6,705	Ex-miners with/out pneumoconiosis only; smoking data incomplete
		SIR = 0.80 CI 0.39–1.48^23^	N = 23,630	Between the years 1973–1992
		RR = 1.87 CI 0.87–4.02^41^	N_c_/N_con_ = 13, 22	Excess risk of bladder cancer among smokers with the job title of coal miner
Bone	No	SIR = 1.67^23^	N = 23,630	Between the years 1973–1992; no confidence interval (observed <5); specifically bone
		SMR = 99 CI 11–345^27^	N = 3,790	Coal miners with abnormal chest x rays; Specifically bone cancer
Brain	No	SIR = 1.05 CI 0.57–1.76^23^	23,630	Between the years 1973–1992
		SMR = 107 CI 46–211^27^	N = 3,790	Coal miners with abnormal chest x rays
Colon/Rectum	No	SIR_w_ = 0.96 CI 0.56–1.55^28^	N_w_ = 2,158	Ex-miners with pneumoconiosis only; smoking data incomplete
		SIR_w/o_ = 0.88 CI 0.62–1.20^28^	N_w/o_ = 6,705	Ex-miners with/out pneumoconiosis only; smoking data incomplete
		SIR_Rec_ = 0.83 CI 0.46–1.83^23^	N = 23,630	Between the years 1973–1992; Specifically rectal cancer
		SIR = 1.00 CI 0.66–1.45^23^		Between the years 1973–1992
		SMR = 95^44^	N_c_/N_con_ = 1,796;4,022	1987–1994
		RR = 0.8 CI 0.3–2.3^44^		1987–1994; relative risk for dying of colon cancer; adjusted for age and smoking habits
Digestive/Gastric/Stomach	Yes	SMR (not calculated) p≈0.05^25^	N = 24736	Chi-square analysis by age and level of dust exposure show increased risk with increased dust
		SMR = 147.5 CI 122.3–176.3, P<0.01^27^	N = 3790	Significant only those w/no/mild pneumoconiosis; inverse pneumoconiosis grade and cancer
		OR_w/smokingCCC (>30)_ = 3.52 CI 1.34–9.28^29^	N_c_/N_con_ = 46	Odds ratio for risk of gastric cancer; controlled for smoking more than 30 years, and used two study designs: conventional and matched case-control
		SIR = 123.2–140.0^26^	N = 17,820	All three time frames (1959–2006)
		OR_w/smoking (>30_) = 3.52 CI = 1.11–11.7^30^	N_c_/N_con_ = 46, 138	Odds ratio for risk of gastric cancer; controlled for smoking
		OR = 11.8 CI 1.36–103^36^	N_c_/N_con_ = 354	OR for risk of gastric cancer; not adjusted for smoking, but for professional status and diet
	No	SIR_w_ = 1.08 CI 0.50–2.05^28^	N_w_ = 2,158	Smoking data incomplete
		SIR_w/o_ = 1.15 CI 0.72–1.74^28^	N_w/o_ = 6,705	
		SMR = 0.91 p>0.05^24^	N = 8,878	Exposure to coal mine dust
		SMR = 75 CI 46–114^21^	N = 8,899	U.S. miners initially examined 1969–1971; 22–24 year follow-up
		OR_w/smoking (<30)_ = 0.55 CI = 0.15–1.99^30^	N_c_/N_con_ = 46, 138	Odds ratio for risk of gastric cancer; smoking less than 30 years
		OR_w/o smoking_ = 1.55 CI = 0.76–3.17^30^		Odds ratio for risk of gastric cancer; Controlled for smoking
		SIR = 0.70 CI 0.28–1.44^23^	N = 23,630	Between the years 1973–1992
		SMR = 105^44^	N_c/_N_con_ = 1,796;4,022	1987–1994
		RR = 1.6 CI 0.7–3.8^44^		1987–1994; relative risk for dying of stomach cancer; adjusted for age and smoking habits
		RR_M_ = 1.55,0.78.0.83 CI 0.72–3.3, 0.39–1.56, 0.37–1.89^45^	N_c/_N_con_ = 178,178,138	Relative risk for stomach cancer; males; Controls were digestive cancer, heart disease, and neighborhood respectively
		RR_F_ = 2.14,1.5,1.67 CI 0.87–5.26,0.67–3.34,0.73–3.81^45^		Females; Controls were digestive cancer, heart disease, and neighborhood respectively
		RR = 1.7 CI = 0.8–3.6^32^	N_c_/N_con_ = 95, 190	Had an allowance for diet; manual work in dusty industry
		RSMR = 0.98 CI 0.36–2.11^19^	N = 4,578	Attempted to adjust for healthy worker selection effects
		OR_w/smokingMCC (>30)_ = 6.0 CI 1.26–28.54^29^	N_c_/N_con_ = 46	Odds ratio for risk of gastric cancer; Controlled for smoking and used two study designs: conventional and matched case-control. Also used different controls for matching (reported are other cancer controls)
		OR_w/o smokingCCC_ = 1.55 CI 0.85–2.83^29^		
		OR_w/smokingCCC_ = 0.55 CI 0.19–1.62^29^		
		SMR = 125, p = 0.65^22^	N = 7,939	30 year follow-up
		OR 1.15 CI 0.89–1.47^42^	N_c_/N_con_ = 683	Odds ratio for risk of gastric cancer; not adjusted for smoking
		SIR = 0.57 CI 0.26–1.09^23^	N = 23630	Between the years 1973–1992
Kidney	No	SIR_w_ = 1.07 CI 0.58–1.82^28^	N_w_ = 2,158	Ex-miners with pneumoconiosis only; smoking data incomplete
		SIR_w/o_ = 0.66 CI 0.43–0.97^28^	N_w/o_ = 6,705	Ex-miners with/out pneumoconiosis only; smoking data incomplete
Larngeal/Hypopharyngeal	Yes	OR = 2.1 CI 1.1–4.1^34^	N_c_/N_con_ = 528, 305	OR for risk of laryngeal/hypopharyngeal cancer; males; age/drinking/smoking adjusted
		OR = 2.0 CI 1.0–3.8^34^		Males; age/drinking/smoking/education adjusted
	No	SIR = 1.02 CI 0.37–2.21^23^	N = 23,630	Between the years 1973–1992
Leukemia/Aleukemia	No	SIR = 0.42 CI 0.14–0.98^23^	N = 23,630	Between the years 1973–1992
		SMR = 99 CI 57–158^27^	N = 3,790	Coal miners with abnormal chest x rays
Liver	Yes	SMR = 266^44^	N_c_/N_con_ = 1,796;4,022	1987–1994
Lung/Trachea/Bronchus/Respiratory	Yes	SIR = 115.7^26^	N = 17,820	Only one time frame of three (1990–2005; 1959–2006)
		SIR_w_ = 2.21 CI 1.75–2.76^28^	N_w_ = 2,158	Ex-miners with pneumoconiosis only; smoking data incomplete
		SIR_w/o_ = 0.87 CI 0.70–1.06^28^	N_w/o_ = 6,705	Ex-miners with/out pneumoconiosis only; smoking data incomplete
		OR = 2.7 CI 1.3–5.6^37^	N_c_/N_con_ = 260	Odds ratio for risk of lung cancer; adjusted for subtype of fuel
		OR = 3.8 1.4–10.3^37^		Adjusted for subtype of fuel; worked 10 or more years as a coal miner
	No	OR _w/o smoking = _1.42 CI 0.70–289^30^	Nc/Ncon = 46, 138	OR for risk of lung cancer; controlled for smoking
		OR _w/smoking (>30) = _2.25 CI 0.92–5.49 ^30^		OR for risk of lung cancer; smoking less than 30 years
		OR _w/smoking (<30)_ = 0.27 CI 0.00–1.29 ^30^		OR for risk of lung cancer; smoking more than 30 years
		OR_1∶1/living_ = 0.87 CI 0.52–1.45^31^	N = 317	1∶1 and 2∶1 Matching both matched on age at death and age of living miners; OR’s are risks for longer versus shorter underground coal mining
		OR_1∶1/death_ = 1.18 CI 0.86–1.62^31^		
		OR_2∶1/death_ = 0.89 CI 0.66–1.20^31^		
		OR_2∶1/living_ = 0.80 CI 0.48–1.32^31^		
		SIR = 0.74 CI 0.50–1.06^23^	23,630	Between the years 1973–1992
		OR = 1.74 CI 0.71–4.25^39^	N_c/_N_con_ = 42	Deaths 1968–1974
		OR = 0.95 CI 0.65–1.38^4^	N_c/_N_con_ = 381	Odds ratio for risk of lung cancer; not adjusted for smoking
		OR_W1_–_9_ = 1.9 CI 0.9–3.9^43^	N_Tc/_N_Tcon_ = 3,792;1,966	Odds ratio for risk of lung cancer; controls are Colon/Rectrum cancer; white males
		OR_W10+ = _1.9 CI 0.9–4.2^43^		Controls are Colon/Rectum cancer; white males
		OR_B1_–_9_ = 4.1 CI 0.9–18.8^43^		Controls are Colon/Rectum cancer; black males
		OR_B10+ = _3.1 CI 0.5–18.0^43^		Controls are Colon/Rectum cancer; black males
		OR_1,2,3_ = 1.23,1.25,1.23 CI 0.79–1.90^38^	N_c/_N_con_ = 1,004	1988–1993; odds ratio for risk of lung cancer; 1 = not adjusted, 2 = adjusted for smoking, 3 = adjusted for smoking/asbestos
		SMR = 152^44^	N_Tc/_N_Tcon_ = 1,796;4,022	1987–1994
		RR = 1.6 CI 0.8–3.4^44^		1987–1994; relative risk for dying of lung cancer; adjusted for age and smoking habits
		RSMR = 1.11 CI 0.8–1.51^19^	N = 4,578	Attempted to adjust for healthy worker selection effects
		SMR = 0.77 P<0.05 ^24^	N = 8,878	Exposure to coal mine dust
		SMR = 107 CI 95–119^21^	N = 8,899	U.S. miners initially examined 1969–1974; Specifically lung with trachea/bronchus
		SMR = 105 CI 94–116^21^		U.S. miners initially examined 1969–1974;Specifically respiratory which includes lung, bronchus, and pleura
		SMR = 102 CI 90–115^27^	N = 3,790	Coal miners with abnormal chest x rays
Lymphomas	No	SIR = 1.13 CI 0.90–1.39^23^	N = 23,630	Between the years 1973–1992
		SMR = 133 CI 72–328^27^	N = 3,790	Coal miners with abnormal chest x rays
Melonoma	No	SIR = 1.13 CI 0.90–1.39^23^	N = 23,630	Between the years 1973–1992
		SMR = 118 CI 38–274^27^	N = 3,790	Coal miners with abnormal chest x rays
Mouth/Buccal Cavity/Oral	No	SMR = 16 CI 0–79, P<0.05^27^	N = 3,790	Coal miners with abnormal chest x rays; Specifically a decrease for the buccal cavity & pharynx
		SIR = 1.02 CI 0.49–1.87^23^	23,630	Between the years 1973–1992; specifically lip
		SIR = 0.49 CI 0.21–0.97^23^		Between the years 1973–1992; specifically other
Multiple myeloma	No	SIR = 0.28^23^	23,630	Between the years 1973–1992; no confidence interval (observed <5); specifically multiple myeloma
		SMR = 62 CI 22–133^27^	N = 3,790	Coal miners with abnormal chest x rays; Specifically multiple myeloma
Nasal	Yes	SIR = 160 p<0.01^20^	N = 1,602	England and Wales from 1963–1967
	No	SIR = 0.54^23^	N = 23,630	Between the years 1973–1992; no confidence interval (observed <5)
		SMR = 205 CI 30–714^27^	N = 3,790	Coal miners with abnormal chest x rays
Pancreas	No	SMR = 123^44^	N_c/_N_con_ = 1,796;4,022	1987–1994
		SMR = 71 CI 44–108^27^	N = 3,790	Coal miners with abnormal chest x rays
Prostate	No	SIR = 0.43 CI 0.16–0.94^23^	N = 23630	Between the years 1973–1992
		SMR = 69 CI 49–94^27^	N = 3,790	Coal miners with abnormal chest x rays
Testis	No	SIR = 0.95 CI 0.59–1.45^23^	N = 23631	Between the years 1973–1993
		SMR = 110 CI 12–383^27^	N = 3,790	Coal miners with abnormal chest x rays
Total (combined) Cancers	Yes	SMR = 150^44^	N_c/_N_con_ = 1,796;4,022	1987–1994
		RR = 1.5 CI 1.1–2.1^44^		1987–1994; relative risk for dying of cancer; adjusted for age and smoking habits
	No	SMR = 95 CI 88–101^21^	N = 8,899	U.S. miners initially examined 1969–1974; 22–24 year follow-up
		RSMR = 1.03 CI 0.84–1.25^19^	N = 4,578	Attempted to adjust for healthy worker selection effects
		SIR = 0.82 CI 0.73–0.92^23^	N = 23,630	Between the years 1973–1992
		SMR = 97 CI 90–104^27^	N = 3,790	Coal miners with abnormal chest x rays

**Table 3:** Estimations of cancer risks reported from population (ecological) studies.

Cancer	Increased risk?	Values	N	Caveats
Bladder	Yes	RR_M_ = 1.13 CI 1.03–1.24^48^	N = 126 towns	Relative risk of dying from bladder cancer; towns situated at a distance of 5 km or less form mining installations; Male
	No	RR_B_ = 1.11 CI 1.02–1.20^48^	N = 126 towns	Relative risk of dying from bladder cancer; towns situated at a distance of 5 km or less form mining installations; Both sexes
		RR_F_ = 1.02 CI 0.86–1.22^48^		Relative risk of dying from bladder cancer; towns situated at a distance of 5 km or less form mining installations; Female
Breast	No	R = 0.26^50^	Population-based	Correlation between mining activity and cancer mortality
Colon/Rectum	Yes	RR_B_ = 1.10 CI 1.04–1.16^48^	N = 126 towns	Relative risk of dying from colon cancer; towns situated at a distance of 5 km or less form mining installations; Both sexes
		RR_M_ = 1.10 CI 1.03–1.17^48^		Relative risk of dying from bladder cancer; towns situated at a distance of 5 km or less form mining installations; Male
		RR_F_ = 1.09 CI 1.02–1.17^48^		Relative risk of dying from bladder cancer; towns situated at a distance of 5 km or less form mining installations; Female
Digestive/Gastric/Stomach	No	R = 0.13^50^	Population-based	Correlation between mining activity and cancer mortality
		SMR_males_ = 92_mining_ and 91 _non-mining_^47^; SMR_females_ = 104_mining_ and 86 _non-mining_^47^	10 towns (6 w/mining and 4 w/0 mining)	Examination of excess stomach cancer risk; slight increase amongst females, but no consistent pattern noted
Leukemia/Aleukemia	Yes	RR_B_ = 1.09 CI 1.00–1.19^48^	N = 126 towns	Relative risk of dying from leukemia; towns situated at a distance of 5 km or less form mining installations; Both sexes
	No	RR_M_ = 1.12 CI 1.00–1.25^48^	N = 126 towns	Relative risk of dying from leukemia; towns situated at a distance of 5 km or less form mining installations; Male
		RR_F_ = 1.12 CI 0.99–1.27^48^		Relative risk of dying from leukemia; towns situated at a distance of 5 km or less form mining installations; Female
Lung	Yes	RR_c1_ = 1.21^46^	Population based	Relative risk for lung cancer; Cluster 1; Adjusted for county-level gender, age, and lifetime smoking prevalence
		RR_c2_ = 1.17^46^		Relative risk for lung cancer; Cluster 2; Adjusted for county-level gender, age, and lifetime smoking prevalence
		SMR = 14.3% ±4.6%^52^	N = 3,314 areas	1969–1978; adjusted for smoking; predicted increase in SMR for males
		RR_M_ = 1.08 CI 1.02–1.14^48^	N = 126 towns	Relative risk of dying from lung cancer; towns situated at a distance of 5 km or less form mining installations; Male
		OR = 2.03 CI 1.32–3.13^51^	N = 756	Odds ratio for risk of cancer; controlled for the effect of covariates
		Regression Coefficient 3.72, p<0.036^49^	Population based	Appalachian coal-mining exposure
		R = 0.57, p<0.0001^50^	Population-based	Correlation between mining activity and cancer mortality
	No	RR_B_ = 1.07 CI 1.01–1.13^48^	N = 126 towns	Relative risk of dying from lung cancer; towns situated at a distance of 5 km or less form mining installations; Both sexes
		RR_F_ = 0.97 CI 0.86–1.09^48^		Relative risk of dying from lung cancer; towns situated at a distance of 5 km or less form mining installations; Female
Oral	No	R = 0.07^50^	Population-based	Correlation between mining activity and cancer mortality
Urinary	No	R = 0.08^50^	Population-based	Correlation between mining activity and cancer mortality
Total (combined) Cancers	Yes	R = 0.55, p<0.0001^50^	Population-based	Correlation between mining activity and cancer mortality
		OR = 2.03 CI 1.32–3.13^51^	N = 773	Odds ratio for risk of cancer; residence in coal river; adjusted for age/smoking

The 10 studies comprising category A1 were conducted from the 1950s through 2006 and include anywhere from 1,602 to 24,736 miners, while the 17 studies in category A2 (all case/control except for Une et al which used population split into cohorts) were conducted from the late 1960s through 1994 and are generally smaller in scale with sample sizes ranging from 92 to >16,000 (only 4 exceeded 1,000 individuals). Individual cancer results are shown in Table 2; these studies variously report incidence and/or mortality for 19 cancer sites/categories. Consistent assessment of risk exists for bone, brain, colon/rectum, kidney, leukemia/aleukemia, lymphomas, melanoma, mouth/buccal cavity/oral, multiple myeloma, pancreas, prostate and testis (no increased risk), and liver (increased mortality). Several studies report multiple risk assessments based upon different adjustments, exposures, or populations studied. The 7 studies from category B (ecological/cross-sectional) are generally much more recent, using data ranging from 1969–2006, and are generally larger, including, for example, all administrative areas in Japan or Appalachian counties [49], [52]. Individual cancer results are shown in Table 3; these studies variously report incidence and/or mortality for 9 cancer sites/categories. Consistent assessment of risk is found for breast, digestive/gastric/stomach, oral and urinary (no increased risk), and colon/rectum and total/combined (increased incidence or mortality). Again, one study here reported differing risks based upon gender.

Table 4 lists all cancer sites for which risks were assessed and reported, and the numbers of studies for each. While the digestive/gastric/stomach and lung/trachea/bronchus/respiratory categories have multiple assessments of both incidence and mortality, 4 cancers lack any assessment of incidence risk (breast, liver, pancreas, urinary), 2 lack any assessment of mortality risk (kidney and laryngeal/hypolaryngeal), and 11 have only a single study reporting risk assessment of incidence or mortality.

**Table 4:** Listing of cancers specifically assessed by selected studies* and the number reporting incidence and mortality for each.

Cancer site	Incidence		Mortality	
	# showing increase	# showing no increase	# showing increase	# showing no increase
Bladder	2	3	1^1^	2^1^
Bone	0	1	0	1
Brain	0	1	0	1
Breast	0	0	0	1
Colon and Rectum	0	2	1	2
Digestive/Gastric/Stomach	4	7	2	8
Kidney	0	1	0	0
Laryngeal/hypolaryngeal	1	1	0	0
Leukemia/aleukemia	0	1	1^2^	2^2^
Liver	0	0	1	0
Lung/Trachea/Bronchus/Respiratory	6	6	3^3^	8^3^
Lymphomas	0	1	0	1
Melanoma	0	1	0	1
Mouth/Buccal Cavity/Oral	0	1	0	2
Multiple myeloma	0	1	0	1
Nasal	1	1	0	1
Pancreas	0	0	0	2
Prostate	0	1	0	1
Testis	0	1	0	1
Urinary	0	0	0	1
Total (combined) cancers	1	1	3	3

* It is noted that multiple studies reported on more than a single cancer site and the total does not therefore equal 34.

1 A study reported increased risk for males but no increase for females.

2 A study reported increased risk for total population, but no increase when examined by gender.

3 A study reported increased risk for males but no increase for females.

## Discussion

We identified 34 studies published since 1980 specifically examining the increased risk of cancer associated with coal mining and associated activities. Twenty-seven of these explicitly examined coal miners/coal mining as an occupational cohort or risk factor. Coal miners as a group have long been studied for adverse health outcomes, and liver was the only cancer site for which only an increased risk was reported (mortality; single study). There were only 7 studies found specifically examining the association between proximity to coal mining activities and cancer risk in the general population, and unequivocal increased risk was found for colon/rectum (mortality) and total (combined) cancer (incidence and mortality). However, increased risks for population subsets were also unequivocally reported for bladder (males, mortality) and leukemia/aleukemia (combined genders, mortality). While the population studies are generally more likely to report increased risk, this may be attributed in part to publication bias, or perhaps the tendency for coal mining regions to have high poverty rates. Some areas with both high cancer rates and coal mining activity also face increased smoking, overweight, and other cancer risk factors [49], [51]. Overall, it is difficult to ascertain cancer risk associated with exposure to coal mining, due to the contradictory results of research examining commonly studied cancer sites, the paucity of studies examining other sites, and the weaknesses inherent in cross-sectional, population-level studies.

However, given the wide scale and extent of coal mining in the US, the potential exposure of large populations to mining activities, the potential for significantly increased risk of cancer incidence and mortality, and the perhaps modifiable nature of the exposures, our position is that a closer and more rigorous examination of cancer risk associated with coal mining exposure is warranted based upon the Hill criteria [53]. These criteria were originally developed for application to infectious disease, and so some may not be as suitable (e.g. **specificity**) or easily evaluated (e.g. **temporality**) when applied to cancer. For example, the cohort studies included here contribute data concerning the temporal relationship between mining activity and cancer, but this is more problematic for the case-control studies where there may be biases in remembering exposure types, dates and duration. There is some evidence that increasing exposure leads to increased cancer risk (**dose-response relationship**). In miners, this was seen in increased risk with longer exposure (years mining) [27], [37]. Of the seven ecological/cross-sectional studies we identified, six of them showed increased cancer risk (**strength of association**), with calculated RR and OR significantly increased for 7 specific cancers reported in 7 studies, as well as total cancer reported in 3 studies (Table 3). While the data from ecological/cross-sectional studies are sparse, they fairly consistently show an increased risk of cancer in association with residence near coal mining (**consistency**). However, only the results regarding colon and rectal cancers and total cancers are unequivocal, with some cancer risks specific to location or gender [48].

**Plausibility** is perhaps the strongest criteria here. Whong et al showed that the nitrosation of coal extracts via acid exposure increased mutagenic activity – possibly contributing to the observed increase gastric cancer risk [54]. Studies of coal dust exposure (not definitively related to coal mining and thus not previously included) report increased risk of lung cancer [55]–[57]. As coal may contain high amounts of carcinogens such as silica dust, polyaromatic hydrocarbons, cadmium, arsenic and others, it is plausible to consider a link between coal dust inhalation into the lungs and subsequent cancer [58], [59]. Indeed, studies have shown increased lung cancer risk in homes using coal as a fuel for cooking or heating and the IARC has determined that indoor emissions from the household combustion of coal are carcinogenic [60]–[62]. One study has even detailed chronic poisoning effects from burning coal containing high levels of arsenic (100–9,000 ppm) [63]. Other findings suggesting that exposure to coal components increases risk include increased malignancy/cell proliferation of kidney cells exposed to large molecular weight compounds mobilized from lignite beds [64], greatly increased levels of crystalline silica in coal in areas of high lung cancer [65], association between high female lung cancer mortality and high-silica coal mine proximity [59], and increased DNA mutations found in mice and rats living in coal mining areas compared to non-exposed controls [66]. Finally, there are studies showing that coal mining contaminates the surrounding air and water [67]–[70].

Taken together, the concern that exposure to coal mining may result in increased risk of cancer is reasonable, and fits with models of exposure, pathogenicity, and outcome (**coherence**). Minerals associated with coal deposits often include human carcinogens (e.g. As, silica). Individuals may be exposed to them in multiple routes (e.g. inhalation, ingestion). There are biologically plausible pathways whereby exposure may result in cancer genesis/promotion. And finally there are population-level studies showing that residing near coal mining increases an individual’s cancer risk.

The last two of Hill criteria, **alternate explanations** and **experiment**, are not likely to add or detract from our position. For example, it is well known that smoking is the greatest risk factor for lung cancer and may at times be poorly captured or adjusted for, and second-hand smoke exposure even more difficult to ascertain. It is likely that there will be few cancers for which alternative explanations do not contribute.

There are limitations to this study. For example, included studies encompass a wide time frame (1980 through 2012) and were conducted in 12 countries. It is likely that there are substantial variations in culture, as well as exposure and safety mechanisms across time and location, which are not accounted for. Complicating the direct comparison of cancer risk is the differences in how cancer sites were described (e.g. ‘gastric’ and ‘stomach’), frequently with no clear definition. We therefore grouped studies together as seemed appropriate, but may be in error. Some studies did not include measures of confidence or significance, limiting our ability to objectively determine if there was in fact increased risk observed. To be conservative, we assumed non-significance in the absence of other confidence or significance data, but this may be in error. For the ecological studies, their cross-sectional nature limits their ability to impute strong association. Finally, the diversity of data collected, cancer sites reported and populations studied precludes the utility of meta-analysis, making our study much more qualitative in nature.

Given the increasing use of coal for energy production in the US, the large numbers of individuals potentially exposed to agents associated with coal mining activities, the equivocal nature of existing studies, and the plausibility for exposure to increase cancer risk, further investigation is needed. Specifically, the available data indicate that there is a need to purposefully and prospectively examine the risk of cancer to the surrounding population from coal mining activity. At this point little is known concerning routes, duration and timing of exposures; which specific agent(s) may be associated with increased cancer risk; or the population at risk in terms of residential proximity. Furthermore, while much study has been made in general concerning personal attributes and behaviors which may aggravate/mitigate exposure and cancer risk, it is unknown how these interact with exposure to coal mining activities. Such items need to be investigated if effective interventions are to be designed, implemented, and evaluated. Such studies, however, would need to be large in scale and long-term. One occupational study, for example, showed lung cancer risk lagging exposure by 15 years.^29^ Recognizing that such studies are likely quite expensive and perhaps infeasible, we propose that interim markers of exposure or increased cancer risk be developed, validated and used as proxies.

## Supporting Information

Checklist S1 **PRISMA checklist.**
(DOC)
